# Supplementary material for: The genome sequence of the bronze bream, Abramis brama (Linnaeus, 1758) (Cypriniformes: Leuciscidae)
Source: Wellcome Open Res. 2025 Jul 28;10:371. [Version 1] doi: 10.12688/wellcomeopenres.24606.1 (PMC12441676; doi:10.12688/wellcomeopenres.24606.1)
Supplement: Peer review history

# Reviewer report: Reviewer response for version 1

**DOI:** 10.21956/wellcomeopenres.27116.r130958

**Published:** 2025-09-16

**Authors:** Karim Gharbi

**Affiliations:** 1 Earlham Institute, Norwich, England, UK

This data note describes a high-quality reference assembly of the common bream (Abramis brama) genome, with Ensembl annotation generated as part of the Darwin Tree of Life Project. The note includes a general overview of the species biology followed by a standardised genome report. The introduction is informative and the methods comprehensive. Assembly statistics and assessment are consistent with a high-quality, reference-grade genome assembled to chromosome-level scaffolds/pseudo-molecules. Overall, this study represents an important contribution to biodiversity genomics.

 While not critical, the report contains a number of omissions and inconsistencies, which the authors may want to rectify:

 1. The species is referred to both 'bronze' and 'common' bream throughout the text. I'd recommend picking one to avoid confusion. 

 2. The sex of the sampled individual is not described. If this is known, it should be added. If it's unknown, it should be stated. 

 3. The tissue for HMW extraction is described as 'gill animal'. This should probably read 'gill' or 'animal's gill'.

 4. Kits and instruments are described differently between sections with regard to the manufacturer. This should be standardised, ideally. 

 5. Yield and purity of the RNA extraction is not provided, while it is for HMW DNA. 

 6. The number of PCR cycles for HiC library preparation is given as a range. This should be provided as a fixed value for this sample. 

 7. The sequencing chemistry used for Illumina sequencing is missing.

 8. Basecelling software versions for Sequel IIe and NovaSeq are missing. 

 9. BUSCO metrics in the text, including % identified, % single, and % duplicated, seem to be incorrect and do not align with those in Figure 5. 

 10. The average transcript length is one order of magnitude larger than the average calculated from the corresponding fasta file in Ensembl.

Are sufficient details of methods and materials provided to allow replication by others?

Yes

Is the rationale for creating the dataset(s) clearly described?

Yes

Are the datasets clearly presented in a useable and accessible format?

Yes

Are the protocols appropriate and is the work technically sound?

Yes

Reviewer Expertise:

Genomics and bioinformatics

I confirm that I have read this submission and believe that I have an appropriate level of expertise to confirm that it is of an acceptable scientific standard, however I have significant reservations, as outlined above.

# Reviewer report: Reviewer response for version 1

**DOI:** 10.21956/wellcomeopenres.27116.r130956

**Published:** 2025-09-02

**Authors:** Qiong Shi

**Affiliations:** 1 College of Life Sciences and Oceanography, Shenzhen University, Shenzhen, Guangdong, China

The authors performed whole-genome sequencing and constructed a chromosome-level genome assembly for the bronze bream. The mitochondrial genome of this fish was also assembled. Generally speaking, the overall writing of this manuscript is ok, and numerous data were generated to support the main conclusions. However, major revisions are still required before acceptance for indexing.

 1. It is recommended to change the title of this data note as follows:

 A chromosome-level genome assembly of the bronze bream ( *Abramis brama*).

 2. Background:

 (1) Lines 5-6: Change “sexually” to “sexual”.

 (2) Please unify the English name of *Abramis brama* (common bream or bronze bream).

 3. Methods:

 (1) The first line of page 4: What is “gill animal”? It also appears in the Table 1.

 (2) Why didn’t you use muscle for the PacBio HiFi sequencing (as the Hi-C)?

 4. Results:

 (1) Under “Assembly quality metrics” (page 6): In lines 4-7, some data are obviously wrong.

 (2) Figure 3: Please adjust this figure, since the current version is unclear.

 5. Data availability: Make sure to release all the genomics data before acceptance of this manuscript.

Are sufficient details of methods and materials provided to allow replication by others?

Yes

Is the rationale for creating the dataset(s) clearly described?

Yes

Are the datasets clearly presented in a useable and accessible format?

Partly

Are the protocols appropriate and is the work technically sound?

Yes

Reviewer Expertise:

Fish genomics and molecular breeding

I confirm that I have read this submission and believe that I have an appropriate level of expertise to confirm that it is of an acceptable scientific standard, however I have significant reservations, as outlined above.

# Reviewer report: Reviewer response for version 1

**DOI:** 10.21956/wellcomeopenres.27116.r129974

**Published:** 2025-09-02

**Authors:** Jayan Duminda M Senevirathna

**Affiliations:** 1 Uva Wellassa University, Badulla, Uva Province, Sri Lanka

The article is well-written and provides valuable insights into the genome of an important fish species. The study holds significant relevance and contributes to the broader understanding of this species’ biology.

 The scientific name of the species should be italicized in accordance with standard scientific writing conventions.

 It would be beneficial to include information on the species’ sexual dimorphism and special adaptations to its habitat as part of the background. This will provide readers with a stronger biological context for the genomic study.

Are sufficient details of methods and materials provided to allow replication by others?

Yes

Is the rationale for creating the dataset(s) clearly described?

Yes

Are the datasets clearly presented in a useable and accessible format?

Yes

Are the protocols appropriate and is the work technically sound?

Yes

Reviewer Expertise:

Aquatic Biotechnology and Genetics

I confirm that I have read this submission and believe that I have an appropriate level of expertise to confirm that it is of an acceptable scientific standard.
